# What Influences Patients Readiness for Discharge: The Case of Total Knee Arthroplasty: A Cross-Sectional Study

**DOI:** 10.1155/2024/8032254

**Published:** 2024-04-01

**Authors:** Na Li, Paulo Moreira, Manjie Guo, Simeng You, Brayal Dsouza, Hong Ji

**Affiliations:** ^1^Nursing Department, The First Affiliated Hospital of Shandong First Medical University & Shandong Provincial Qianfoshan Hospital, Jinan, Shandong, China; ^2^Henan Normal University, School of Social Affairs, Xinxiang, Henan, China; ^3^Atlantica Instituto Universitario, Healthcare Management, Oeiras, Portugal; ^4^International Healthcare Management Research and Development Center (IHM-RDC), The First Affiliated Hospital of Shandong First Medical University & Shandong Provincial Qianfoshan Hospital, Jinan, Shandong, China; ^5^School of Nursing and Rehabilitation, Shandong University, Jinan, Shandong, China; ^6^Department of Social and Health Innovation, Prasanna School Of Public Health, Manipal Academy of Higher Education, Manipal, India; ^7^Kasturba Hospital, Manipal, India

## Abstract

**Background:**

Evidence has suggested that most clinical staff use clinical laboratory indicators to determine discharge times, while paying little attention to patients' feelings and needs. Additional research findings have suggested a relationship between patients' self-reported readiness for hospital discharge and postdischarge complication rates, readmission rates, mortality, as well as quality of life. RHD is strongly associated with patient health outcomes. Identifying relevant influencing factors can provide guidance for early individualized interventions by healthcare professionals.

**Design:**

A cross-sectional study.

**Methods:**

During 2022, a total of 320 post-TKA patients were selected for this study. The patients were divided into the low-RHD group (<7 points) and the high-RHD group (≥7 points) according to the mean score of the Readiness for Hospital Discharge Scale (RHDS). Established scales were used to collect patients' information and to adopt univariate and binary logistic regression analysis to screen for independent factors.

**Results:**

In this study, the RHDS score of patients after TKA is 91.90 ± 7.05, of which 12.8% are in the low-RHD group (mean score <7). The binary logistic regression results reveal that age, educational level, postactivity pain, self-efficacy, and family care have to be considered risk factors generating low-RHD in TKA patients.

**Conclusions:**

The present study suggests that over 1/8 TKA patients are not ready at the time of discharge. Physicians and nurses can improve patients' RHD by reducing postactivity pain and improving self-efficacy during their rehabilitation period. *Relevance to Clinical Practice*. The results of this study can help physicians and nurses early identify high-risk patients with low RHD and provide them with individualized interventions. In addition to this, it is important that nurses use RHDS to assess the readiness of TKA patients before they are discharged from the hospital.

## 1. Introduction

As the aging and obese population increases, the number of total knee arthroplasty (TKA) shows a yearly upward trend [1–3]. The annual number of TKA in China has also grown rapidly from 50,000 cases a decade ago to nearly 400,000 cases, with a growth rate of about 27.43% per year, and the number is still rising [4]. It is expected that TKA will become one of the most common surgical operations in the next decade [5].

In response to the healthcare management challenges from increasing number of TKA, hospitals are taking measures to reduce the length of stay of patients [6–8]. However, the TKA patients usually require 3–6 months support for functional exercise [9]. Thus, as shorter hospital stays reduce the amount of time available to prepare patients for discharge, the risk that their readiness may be affected may increase. In addition to this healthcare management challenge, evidence has suggested that most clinical staff use clinical laboratory indicators to determine discharge times, while paying little attention to patients' feelings [10, 11]. Hence, additional research findings have suggested a relationship between patients' self-reported readiness for hospital discharge and postdischarge complication rates, readmission rates, mortality, as well as quality of life [12–14]. Already in 2017, evidence identified readiness for hospital discharge (RHD) as one of the key indicators of patient safety discharge, which include physical stability, adequate support, psychological ability, and adequate information and knowledge [15]. Identifying the current status and factors that affect RHD in patients after TKA can support physicians and nurses to improve and implement early, individualized interventions with positive impacts on the quality of health care. The literature review undertaken exposed the scarcity of studies on RHD in patients with TKA. Also, as people with different diseases tend to register different RHDS scores and different influencing factors [16], looking into the specificity of TKA patients is justified. In this sense, the main objective of this study is to investigate the current status of RHD in TKA patients and to identify influencing factors. The evidence generated in this article aims to provide guidance for physicians and nurses to improve the readiness to hospital discharge in TKA patients.

### 1.1. Theoretical Framework

**Figure 1:**
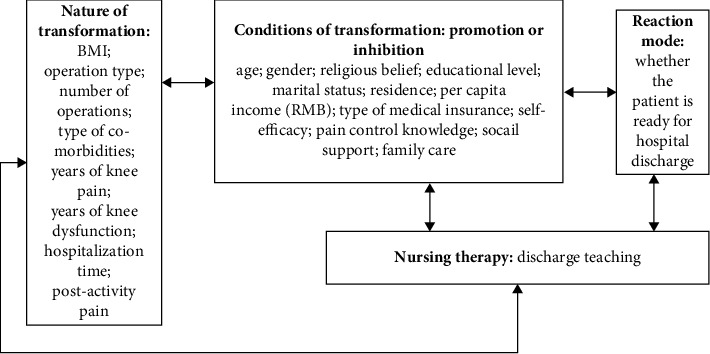
The theoretical framework of the research. Note: BMI = body mass index.

Meleis introduced the concept of transition into nursing in the 1960s, developing the transition theory. Transformation theory mainly comprises the following 4 core notions: the transformed nature, the transformed conditions, the reaction mode, and the care therapy [17]. Based on this, we explore the transformed nature (BMI, type of surgery, etc.), the transformed conditions (age, gender, self-efficacy, etc.), and nursing therapy (discharge teaching) on the reaction mode (the RHD of post-TKA patients) (more details in Figure 1).

## 2. Methods

### 2.1. Study Design and Setting

This is a cross-sectional study. From January to May 2022, post-TKA patients from orthopedic departments are conveniently selected for this study in three third-grade A hospitals in Jinan, Shandong Province. In regression analysis, one should have a sample size of 5–10 times the independent variables. There are 25 independent variables involved in this study, and considering 10% ineffective questionnaires, thus requiring at least 275 patients.

### 2.2. Participants

Three hundred and twenty patients after TKA are recruited for this study. Participants are included according to the following criteria: (a) patients after TKA; (b) from Shandong Province; (c) good at communicating with others; and (d) willingly join the research and give written consent. Participants are excluded according to the following criteria: (a) with a history of mental illness and (b) develop complications after surgery such as deep vein thrombosis and infection.

### 2.3. Data Collection

The collection of data is carried out by two graduate students, and both of them have completed 6 months of internship in the Orthopedic Department. The survey is conducted on the day of the patient's discharge, in a quiet room, and takes approximately 20 minutes per patient. Considering that some of the participants are older and have a lower educated level, this research uses a one-on-one question-and-answer format. That is, the graduate student asks the patients about the questionnaire entries and then presents their answers objectively on the questionnaire.

### 2.4. Instruments

Based on Galvin et al.'s conceptual analysis of RHD, physical (disease-related information), support (social support and family care), psychological ability (self-efficacy), and knowledge (pain control knowledge) are factors that influence a patient's RHD. In addition to this, the scales used in this study are also chosen according to Meleis' transition theory. In summary, we have used the following questionnaires and scales to assess data relating to patients after TKA.

#### 2.4.1. General Information Questionnaire

It is developed by members of the group based on a review of the literature and consultation with clinicians, including demographic information and disease-related information.

#### 2.4.2. Readiness for Hospital Discharge Scale (RHDS)

The RHDS was developed by Weiss and Piacentine [18] based on Meleis' transition theory. We use a Chinese version of Lin et al. [19] for this study. The scale consists of 12 questions, each scored 0–10. A mean score of entries on the scale will be used to determine a patient's RHD, with a score of <7 indicating low RHD and ≥7 indicating high RHD [20]. There are 3 dimensions in total, which are “Personal Status,” “Adaptive Capacity,” and “Expected Support.” The Cronbach's alpha coefficients for the overall and three dimensions are 0.883, 0.821, 0.851, and 0.778, respectively. The validated Chinese version of the Readiness for Hospital Discharge Scale (RHDS) has been used in previous studies [11, 21]. In addition, there was a pretest. This study used 30 patients in a presurvey, and the results showed that they could understand and answer the questionnaire well.

#### 2.4.3. Visual Analogue Scale (VAS)

The VAS is a widely used pain measurement tool to assess the intensity of a patient's pain currently or over 24 hours [22]. It is a 10-cm long straight line with a score ranging from 0 to 10, of which a higher score indicates stronger pain [23]. In this study, it is used to assess the postactivity pain in TKA patients. The Chinese version of this scale has been widely used in studies [24, 25].

#### 2.4.4. Quality of Discharge Teaching Scale (QDTS)

The QDTS is developed by Weiss et al. [26] to measure the quality of discharge teaching as perceived by patients, which includes three dimensions, a total of 24 questions with 0–10 points each. Higher scale scores indicate better quality of discharge teaching for patients. The overall Cronbach's alpha coefficient is 0.863. The pretest of the Chinese-translated version was undertaken by many researchers [27, 28].

#### 2.4.5. Self-Efficacy for Rehabilitation Outcome Scale (SER)

The SER is developed by Waldrop et al. [29], which includes 12 questions of 0–10 marks each. The scale has been Sinicized and extensively adopted among the Chinese populace [30]. This study uses a total SER score to assess the beliefs of TKA patients in applying adaptive behavior during the rehabilitation period, with higher scores associated with greater self-confidence. The overall Cronbach's alpha coefficient is 0.908.

#### 2.4.6. Pain Control Knowledge Questionnaire

The Pain Control Knowledge Questionnaire, developed by Wen Mei, Sichuan University, China, in 2007 [31], included 8 questions of 1–5 marks each. The researcher has obtained access to the scale via Email. The higher the score, the greater the patient's knowledge of pain control.

#### 2.4.7. Social Support Rating Scale (SSRS)

We use the SSRS developed by Xiao [32] to assess the current status of patients' social support. This scale has 10 items with a score range of 12–66, and the higher the total score, the better the social support. The overall Cronbach's alpha coefficient is 0.720.

#### 2.4.8. Family APGAR Scale

The scale, designed by Smilkstein, consists of 5 items, each with a score of 0–2 [33], which has been used in knee disease patients and has good reliability and validity [34]. In this study, we use the total score of the scale to assess the patient's subjective satisfaction with family functioning. The pretest of the Chinese-translated version was undertaken in a variety of populations [35, 36].

### 2.5. Data Analysis

We use SPSS (version 26.0) for statistical description and statistical analysis. Numbers and percentages are used to describe demographic information and disease-related information. Continuous variables are described by the mean ± standard deviation (SD) if they conform to a normal distribution, and if not, by the median and interquartile range. An independent samples *t*-test, chi-square test, and Fisher's exact test are used to compare the differences between the two groups. A binary logistic regression is used to explore the factors affecting post-TKA patients' RHD, with the model applying a forward stepwise likelihood ratio. The significance level of the hypothesis test is set at *α* = 0.05 (two sides).

Before the binary logistic analysis, a covariance diagnosis was carried out using SPSS 26.0 software, which showed that the variance inflation factor (VIF) values ranged from 1.114 to 3.391. In addition, the aim of this study is to investigate the several factors that influence the readiness of post-TKA patients for discharge rather than to analyse the effect of a particular variable on their RHD.

### 2.6. Ethical Considerations

This study has received approval from the Ethics Committee of the School of Nursing and Rehabilitation, Shandong University (2022-R-021). Before the study begins, the investigators obtained informed consent from the patients and assure them of the confidentiality of the study data.

## 3. Results

In this study, we collect effective data from 320 patients, with 41 (12.8%) patients with a mean score of less than 7 on the RHDS. The age of the patients ranges from 50 to 88 years, and the mean age is 66.49 years (SD: 7.02). More than three quarters of them are women. The majority of patients are nonreligious (93.4%), married (80.6%), overweight or obese (80%), and with comorbidities (74.1%). Over 85% of the patients have their first TKA and have a unilateral replacement. The results of the univariate analysis shows that age, education level, marital status, per capita income, number of comorbidities, hospitalization time, postactivity pain, quality of discharge teaching, self-efficacy, pain control knowledge, social support, and family care are independent factors for RHD in patients with TKA (*P* < 0.05), more details in Table 1.

The patients' scores for total RHDS and scores for each dimension are shown in Table 2. First, we diagnose that there is no multicollinearity between variables which are statistically significant for univariate analysis and then perform a binary logistic regression analysis (forward: LR method). The results show that age, postactivity pain, education level, self-efficacy, and family care are independent factors, more details in Table 3. The Hosmer–Lemeshow goodness-of-fit test suggests that the overall model fit is adequate (*χ*^2^=2.736, *P*=0.950). At the same time, the Nagelkerke *R*^2^ reveal that the logistic regression model explains 69.0% of the variance.

The average length of stay for post-TKA patients was 5–7 days in the three hospitals.

## 4. Discussion

The results of this study show that the mean RHDS score of patients after TKA is (7.66 ± 0.59), an intermediate level. In this research, TKA patients have lower RHD than those medical and surgical patients who had a mean RHDS score of (8.62 ± 1.47) [37]. An analysis of the reasons for this may be as follows: on the one hand, as an invasive procedure, post-TKA patients often have pain and functional limitations [38]. Also, even up to 1/5 of the patients suffer from a fear of movement and a severe reduction in mobility [39], which affects their functional recovery. On the other hand, compared to other surgical operations, like hip arthroplasty, TKA patients require long-term rehabilitation at home [40], which may increase their discharge uncertainty and decrease their RHD. Of the three dimensions of RHDS, adaptive capacity has the lowest score and expected support has the highest score, a fact that is exactly the opposite of what is found in earlier studies [13]. The reason for this result may be connected to the traditional Chinese culture where family members take the initiative to provide patients with living support, encouragement, and favorable conditions for functional exercise. The results of Wang et al.'s study of cancer survivors indicate that Chinese collectivist-oriented culture promotes social harmony and makes it easier for patients to express their inner feelings and thus receive support [41]. On the contrary, low scores on adaptive capacity indicate that patients lack confidence in their ability to recover at home and care for themselves after surgery.

The results of binary logistic regression in this research suggest that the older the age, the worse the patient's RHD (OR = 1.187), which is consistent with previous studies [42]. Older patients are more likely to have poorer health and more comorbidities, which could lead to difficulties in restoring their quadriceps strength and prolong recovery time. So, they are more likely to have a low RHD at the time of discharge. This suggests that doctors and nurses should pay more attention to older patients, formulate comprehensive rehabilitation plans through multidisciplinary consultation, and clarify their disease character. In addition to this, doctors and nurses should improve the patient's physical condition through nutrition, medication, and rehabilitative exercises before surgery, thus improving their RHD.

Postactivity pain is a risk factor for low RHD in patients (OR = 2.461). Postactivity pain is an important problem for TKA patients [43, 44]; about 60% of them experience pain after surgery [45]. Pain can trigger a systemic stress response in the patient, affecting the body's autonomy and immune system, causing a range of postoperative conditions and seriously affecting postoperative rehabilitation [46]. Consequently, physicians and nurses should pay more attention to patients' pain problems and actively adopt various methods such as multimode analgesia [47], a cold compress [48], as well as traditional Chinese medicine treatment [49] to alleviate postoperative pain so that patients can be ready for discharge soon.

Patients with high levels of education are more likely to have high RHD after TKA (OR = 0.093), which is in agreement with several previous studies [50, 51]. First, patients with a high educational level can better understand the content of discharge teaching and make full use of health information resources and second, they can effectively communicate with medical staff and get helpful information. So, nurses should focus on those patients whose education level is an elementary school and below and use easy-to-understand language for discharge teaching, avoiding medical jargon. At the same time, a good relationship should be established between the doctor and the patient so that the patients can truly express their inner needs to the doctors and nurses.

Different from previous findings, self-efficacy is a significant factor that affected patients' RHD in the present research (OR = 0.836), rather than the quality of discharge teaching [11, 21, 52]. Self-efficacy, as a cognitive mechanism regulating behavioral activities in the field of rehabilitation, can facilitate the translation of patients' motivation and willingness to engage in activities into the performance of specific activity behaviors [53]. The postoperative rehabilitation for TKA patients will directly affect the outcome of the surgery and is crucial to their ability to gain independent [54, 55]. Therefore, healthcare professionals could construct rehabilitation programs based on Bandura's self-efficacy theory to increase patients' self-efficacy levels and enhance their confidence in overcoming their illness, thereby improving their RHD.

Family care is also an important factor in RHD for people with TKA (OR = 0.344), not social support as in previous findings [13, 56]. Influenced by the traditional Chinese culture of “filial piety,” children will play a supportive role in their parents' later years. Due to the trauma of the surgery, patients undergo physical and psychological changes after TKA, as well as increased dependency. To this end, the nurse should urge the families to give more care and support to the patients. A good family functioning is effective in reducing postoperative stress and strain, increasing the patient's level of hope, speeding up their recovery process and improving RHD. Thus, healthcare professionals can involve family members in the patient's discharge planning and teach them care skills. Moreover, family members can play a supervisory role in the patient's recovery process.

As the evidence generated in this article aimed to provide guidance for physicians and nurses to improve the readiness for hospital discharge in TKA patients, the following are key recommendations for professionals around the world: First of all, physicians and nurses need to pay attention to patients' RHD and include their self-reported RHD as part of the discharge assessment plan. Then, the hospital should form a multidisciplinary discharge service team consisting of orthopedic specialists, anesthetists, dieticians, rehabilitators, and nurses so as to develop a personalized discharge plan for the patient based on their actual situation. Next, the hospital units could improve the quality of discharge teaching for patients by regularly organizing post-TKA nursing knowledge competitions, nursing skills competitions, teaching competitions, etc. It is also important to include patients' families in discharge teaching. Last but not the least, there should be timely and excellent communication between the higher hospital and the community sector regarding the patient's condition and recovery. Through mobile web-based devices, senior doctors can regularly guide community doctors in assessing patients' recovery to reduce their uncertainty after discharge from hospital.

### 4.1. Limitation

This research still has some limitations. First, the subjects of this study are all from Jinan, Shandong Province, with limited extrapolation of results. Second, this study uses a one-on-one question-and-answer format to collect patient data, which may have some reporting bias. In the future, we could carry out multicenter studies to further explore the factors influencing RHD in patients after TKA.

## 5. Conclusions

The RHD of TKA patients is first brought to our attention. The results of this study shows that TKA patients have a moderate level of RHD and over 1/8 of the patients are not prepared at discharge. In particular, age, postactivity pain, education level, self-efficacy, and family care are factors influencing RHD in patients with TKA.

## 6. Implications for Practice

First, the results of this study provide a basis for clinical staff to identify high-risk patients with low RHD and provide them with individualized interventions. Second, it is important that nurses use RHDS to assess the readiness of TKA patients before they are discharged from the hospital. Third, hospital managers should adhere to a “patient-centered” management model and develop personalized discharge services for patients from the start of their visit. Finally, hospitals should work with the community to assign postdischarge rehabilitation plans for patients in the context of their condition in order to reduce their uncertainty after discharge and improve their RHD [57–60].

## Figures and Tables

**Table 1:** Univariate analysis of RHD in TKA patients (*N* = 320).

Variables	Total (*n* = 320)	Low RHD (*n* = 41)	High RHD (*n* = 279)	*t*/*F*/*χ*^2^	*P*
Age (years)^a^	66.49 ± 7.02	72.32 ± 5.47	65.63 ± 6.81	−6.000	<0.001
Educational level, *n* (%)					
Elementary school and below	139 (43.4)	35 (85.4)	104 (37.3)	33.647	<0.001
Junior high school and above	181 (56.6)	6 (14.6)	175 (62.7)		
Marital status, *n* (%)					
Married	258 (80.6)	25 (61.0)	233 (83.5)	11.623	0.001
Divorced or widowed	62 (19.4)	16 (39.0)	46 (16.5)		
Per capita income (RMB), *n* (%)					
<2000	165 (51.6)	30 (73.2)	135 (48.4)	8.791	0.003
≥2000	155 (48.4)	11 (26.8)	144 (51.6)		
Number of comorbidities, *n* (%)					
0	83 (25.9)	3 (7.3)	80 (28.7)	13.859	0.001
1∼2	214 (66.9)	31 (75.6)	183 (65.6)		
≥3	23 (7.2)	7 (17.1)	16 (5.7)		
Hospitalization time (days)^a^	5.83 ± 1.45	6.37 ± 1.73	5.75 ± 1.39	−2.546	0.011
Postactivity pain (points)^a^	3.93 ± 1.39	5.34 ± 0.99	3.72 ± 1.32	−9.335	<0.001
Quality of discharge teaching (points)^a^	143.34 ± 7.43	138.73 ± 10.22	144.02 ± 6.69	3.216	0.002
Self-efficacy (points)^a^	80.68 ± 6.03	72.63 ± 7.23	81.86 ± 4.83	7.918	<0.001
Pain control knowledge (points)^a^	26.77 ± 4.90	24.80 ± 4.88	27.05 ± 4.84	2.773	0.006
Social support (points)^a^	39.55 ± 5.92	34.78 ± 5.30	40.25 ± 5.68	5.807	<0.001
Family care (points)^a^	6.98 ± 1.06	5.93 ± 0.65	7.13 ± 1.02	10.201	<0.001

*Note*. ^a^: mean ± Standard deviation.

**Table 2:** RHDS score for TKA patients (*N* = 320).

Dimension	Number of entries	Score range	Actual score	Average score
Personal status	3	0∼30	23.48 ± 2.32	7.83 ± 0.77
Adaptive capacity	5	0∼50	36.78 ± 3.72	7.36 ± 0.74
Expected support	4	0∼40	31.63 ± 2.51	7.91 ± 0.63
Readiness for hospital discharge	12	0∼120	91.90 ± 7.05	7.66 ± 0.59

**Table 3:** Binary logistic regression analysis of RHD in patients with TKA (*N* = 320).

Variables	*B*	*Wald*	*P*	OR	95% CI
Ages	0.172	10.686	0.001	1.187	[1.071, 1.316]
Postactivity pain	0.901	10.297	0.001	2.461	[1.420, 4.265]
Educational level	−2.375	10.242	0.001	0.093	[0.022, 0.398]
Self-efficacy	−0.179	14.879	<0.001	0.836	[0.763, 0.916]
Family care	−1.068	8.074	0.004	0.344	[0.165, 0.718]
Constant	5.752	1.090	0.296	314.97	

*Note.* Omnibus test of model coefficients. *χ*^2^=147.461, *P* < 0.001; Pseudo-(Nagelkerke) *R*^2^=0.690; Hosmer–Lemeshow. *χ*^2^=2.736, *P*=0.950. CI, confidence interval; OR, odds ratio.

## Data Availability

The data used to support the findings of this study are available from the corresponding authors upon request.
